# Lymph node dissection in lung cancer surgery

**DOI:** 10.3389/fsurg.2024.1389943

**Published:** 2024-04-08

**Authors:** Akshay J. Patel, Andrea Bille

**Affiliations:** ^1^ Department of Thoracic Surgery, Guy’s Hospital, Guy’s and St. Thomas’ Hospital NHS Trust, London, United Kingdom; ^2^ Institute of Immunology and Immunotherapy, University of Birmingham, Birmingham, United Kingdom

**Keywords:** lymph nodal dissection, thoracic surgery, lung cancer, robotic surgery, minimally invasive therapy

## Abstract

Lung cancer, a leading cause of cancer-related death, often requires surgical resection for early-stage cases, with recent data supporting less invasive resections for tumors smaller than 2 cm. Central to resection is lymph node assessment, an area of controversy worldwide, compounded by advances in minimally invasive techniques. The review aims to assess current standards for lymph node assessment, recent data from the surgical era, and the immunobiological basis of how lymph node metastases impact patient outcomes. The British Thoracic Society guidelines recommend systematic nodal dissection during lung cancer resection, without specifying node removal or sampling. Historical data on mediastinal lymph node dissection (MLND) survival benefits are inconclusive, although proponents argue for lower recurrence rates. Recent trials such as ACOSOG Z0030 found no survival difference between MLND and nodal sampling, reinforcing the need for robust staging. While lobe-specific dissection strategies have been proposed, they currently lack consensus. JCOG1413 aims to compare the clinical benefits of lobe-specific and systematic dissection. TNM-9 staging revisions emphasize the prognostic significance of single-station N2 involvement. Robotic surgery shows promise, with trials such as RAVAL, which reported comparable outcomes to video-assisted thoracic surgery (VATS) and improved lymph node sampling. Immunobiological insights suggest preserving key immunological sites during lymphadenectomy, especially for patients receiving adjuvant immunotherapy. In conclusion, the standard lymph node resection strategy remains unsettled. The debate between systematic and selective dissection continues, with implications for staging accuracy and patient outcomes. As minimally invasive techniques evolve, robotic surgery emerges as an effective and low-risk approach to delivering optimal lymph node assessment.

## Introduction

Lung cancer is the leading cause of cancer-related death worldwide (1). Early-stage lung cancer is amenable to surgical resection with good long-term disease control (2). Recent randomized data have shown that lung cancers smaller than 2 cm can be effectively treated with less extensive lung resection, such as segmentectomy or wedge resection, achieving good long-term outcomes (3, 4). Central to any lung resection is lymph node assessment, which is fundamentally important to the pathological staging of lung cancer. The extent of lymph node resection, whether it involves sampling or radical lymphadenectomy (dissection), remains an area of controversy, with different surgical practices reported worldwide. The ongoing advances in minimally invasive surgical techniques, including robotic, video-assisted, or indeed uni-portal strategies have further contributed to the diversity of intraoperative approaches to lymph node assessment. The purpose of this review is to critically appraise the current standard of lymph node assessment, review recent data with respect to the current surgical era, and evaluate the immunobiological basis of lymph node metastases and how this translates to patient outcomes.

## Current standard

The British Thoracic Society 2010 guidelines (5) advocate that the International Association of the Study of Lung Cancer (IASLC) nodal map (6) should be used in the assessment and staging of lymph node disease. Intraoperatively, one should perform systematic nodal dissection in all patients undergoing resection for lung cancer and remove or sample a minimum of six lymph node stations. The guidelines here do not provide any firm indication as to whether lymph nodes should be removed or sampled.

## Sampling vs. dissection

Historical data addressing the survival benefit of mediastinal lymph node dissection (MLND) have largely yielded inconclusive results with no definite oncological benefit observed. Proponents of full lymph node clearance have stated that by removing occult N2 disease, there is a lower chance of recurrence, hence leading to improved disease-free survival (7). However, it is worth noting that the commonest recurrence pattern in N2 disease is in distant anatomical sites (8). The most contemporary data come from the Canadian study, ACOSOG Z0030 trial, which randomized T1–T2 non-small cell lung cancer (NSCLC) patients to either no further lymph node sampling or full MLND after comprehensive mediastinal staging with negative lymph nodes (2R, 4R, 7, and 10R for right-sided lesions and 5, 6,7, 10l for left-sided lesions) (7). This trial showed no difference in median survival between no sampling and MLND (8.1 vs. 8.5 years, *p* = 0.25) and no difference in locoregional and distant recurrence. Five-year disease-free survival rates were also not different between the groups; 69% vs. 68%, *p* = 0.92 respectively. However, occult N2 disease was found in 21 patients in the MLND group. This data was not translatable to higher-stage disease or patients with known pre-op N1/N2 disease. Given that current preoperative staging cannot reliably rule out N2 disease, MLND is still recommended given that there is no increased risk of morbidity or mortality. The current era of neoadjuvant immunotherapy, which is rapidly evolving and indeed improving overall and event-free survival, makes the case for robust staging to properly stratify patients into the correct treatment arms. Furthermore, data from this setting have shown significantly improved outcomes in those patients who incur a pathologic complete response (pCR) (9–12), which is all the more reason to perform MLND to ensure firm ascription of the pCR or major pathologic response (MPR) states. We know from meta-analytical data from 209 patients across six studies that neoadjuvant immunotherapy offers comparable nodal downstaging (ypN0) to that of ypT(MPR) (OR 1.31 95% CI: 0.84–2.05) and results in satisfactory responses in metastatic lymph nodes (13).

Further work is needed to investigate the pathology of lung cancers resected in the neoadjuvant setting, and criteria have been described by Travis et al. (14); uniformity of surgical resection and completeness of tissue removal are likely to be core tenets of enhancing knowledge and thus care.

## Lobe-specific vs. systematic dissection

Lobe-specific dissection has been described by numerous centers worldwide. This technique focuses on the characteristic mediastinal nodal metastasis patterns that occur depending on the primary tumor location and hence advocates only the dissection of specific draining nodal stations. Clinical trial data from Shanghai (15) has shown that in the setting of cT1N0 NSCLC, there is a specific mediastinal lymph nodal metastasis pattern and thus provides credence to the rationale of a selective lymph node dissection strategy.

Studies, predominantly from the East Asian subcontinent, investigating lobe-specific vs. systematic dissection have reported no major differences in strategy with respect to overall survival, occult N2 rate, and postoperative complication (16). However, data from Okada et al. (17) demonstrated a much higher complication rate postoperatively with systematic nodal dissection (17.3% vs. 10.1%, *p* = 0.005), with the most common problem being arrhythmia. Meta-analytical data (18) from 13 studies and 11,522 patients indicated that lobe-specific nodal dissection had favorable overall survival [hazard ratio (HR)] 0.80, 95% CI: 0.73–0.87] but no difference in recurrence-free survival (HR 0.96, 95% CI: 0.84–1.09) compared to systematic nodal dissection. This study concurred that there was a lower rate of postoperative complications in patients undergoing lobe-specific nodal dissection, e.g., chylothorax [risk ratio (RR) 0.54, 95% CI: 0.35–0.85] and arrhythmia (RR 0.74, 95% CI: 0.57–0.97), than those in patients undergoing systematic nodal dissection.

A large retrospective series from Sloan Kettering (19) showed that in 1,667 patients who all underwent systematic nodal dissection, an overall occult pN2 rate of 9% (*n* = 146) was observed. Moreover, of these patients, 16% (*n* = 22) had mediastinal lymph node metastases beyond the lobe-specific lymphatic drainage; hence, the authors advocated for systematic nodal dissection in all stages of lung cancer (19). It is worth noting that half of these patients (*n* = 11) had multi-station N2 and hence would have still been staged as pN2 through lobe-specific dissection. Balancing the risk of postoperative morbidity and the risk of missing occult N2 disease remains an area of contention more so in the setting of cT1a/b cancers.

The Japanese Clinical Oncology Group (JCOG) has commenced recruitment for a randomized trial (JCOG1413) to confirm the clinical benefit of lobe-specific nodal dissection for stage I-II NSCLC. The primary endpoint is overall survival, and the trial has a non-inferiority design compared to systematic dissection in the setting of lobectomy. The secondary endpoints include relapse-free survival, % local recurrence, % regional lymph node recurrence, and adverse events with a plan to recruit 1,700 patients (20).

## Implication of the TNM-9 staging system

Data have been recently accrued for the revision of the nodal status staging descriptors for the new TNM-9 staging system (21). For clinical (c) and pathological (p) nodal status, data from 45,032 and 35,009 patients, respectively, were made available. N0 to N3 status reflects the pathologically distinct groups, and as demonstrated by TNM-8, each progressive strata has significantly worse survival. Further interrogation of the divisions has shown that single-station N2 involvement (N2a) exhibits a better prognosis than multi-station N2 (N2b) in both clinical and pathological classifications. The difference between N2a and N2b was prognostically significant. From the clinical and pathological classifications, HR for death for N2b and N2a was 1.27 (95% CI: 1.13–1.43; *p* < 0.0001) and 1.46 (95% CI: 1.32–1.62; *p* < 0.0001), respectively. This implementation shows that detection of occult N2 even if single station will be of significant prognostic value and the chance of detection may well be higher through systematic dissection. Data from JCOG1413 will help answer this question, particularly in light of the upcoming TNM revisions.

## Minimally invasive surgical advances and implications on lymph node dissection

Robotic surgery has evolved rapidly worldwide in the last 5 years in thoracic surgery. Clear advantages have been reported on a center-specific level, namely, enhanced visualization, the ability to dissect into the mediastinum, segmental resection, and lymph node dissection (22). From a more objective perspective, three randomized trials have directly compared robotic [robotic-assisted thoracic surgery (RATS)] vs. conventional video-assisted [video-assisted thoracic surgery (VATS)] approaches in lung resection surgery, namely, RAVAL, ROMAN, and RVLob (23–27).

The RAVAL trial (24) primarily sought to assess the health-related quality of life measures for patients following RATS lung cancer resection. Patients were randomized into a 1:1 ratio to either RATS or VATS-lobectomy. From 164 patients (RATS: *n* = 81; VATS: *n* = 83), the mean 12-week health utility score was 0.85 (0.10) for RATS and 0.80 (0.19) for VATS (*p* = 0.02). Significantly, more lymph nodes were sampled [10 (8–13) vs. 8 (5–10); *p* = 0.003] in the RATS arm. The incremental cost/quality-adjusted life year of RATS was $14,925.62 (95% CI: $6,843.69, $23,007.56) at 12 months. The authors concluded that RATS is cost-effective and associated with comparable short-term patient-reported health utility scores when compared with VATS-lobectomy. This could well mature further as we learn more about the implications of different lymph node dissection strategies and the advantage that the robotic approach has in this regard.

The ROMAN study (27) conducted a similarly designed trial in the setting of lung cancer; however, the primary outcome measure was the incidence of adverse events including complications and conversion to thoracotomy. The secondary objectives included the extent of lymph node dissection. The trial closed early due to the lack of favor for the robotic arm in terms of the primary outcome measure. Despite finding no difference between the two arms in perioperative complications, conversions, duration of surgery, or duration of postoperative stay, a significantly greater degree of lymph node assessment by the robotic technique was observed in regards to the median number of sampled nodal stations [6, interquartile range (IQR) 4–6 vs. 4, IQR 3–5; *p* = 0.0002], hilar LNs (7, IQR 5–10 vs. 4, IQR 2–7; *p* = 0.0003), and mediastinal LNs (7, IQR 5–10 vs. 5, IQR 3–7; *p* = 0.0001) (27). Similar findings were shown by the RVLob trial (25), in which the RATS group had a significantly higher number of lymph nodes harvested [11 (IQR, 8–15) vs. 10 (IQR, 8–13), *p* = 0.02], a higher number of N1 nodes [6 (IQR, 4–8) vs. 5 (IQR, 3–7), *p* = 0.005), and more nodal stations examined [6 (IQR, 5–7) vs. 5 (IQR, 4–6), *p* < 0.001). Health-related quality of life and pain scores between the two groups were comparable up to 48 weeks postoperatively (24).

Robotic surgery albeit in its infancy has thus far shown utility in lymph node dissection and may well demonstrate utility and lower incidence of postoperative complications with respect to VATS when specifically assessing systematic lymph node dissection. This remains to be further elucidated. Given the recent data highlighting the utility of segmental resection, the robotic approach may further provide intraoperative superiority for intersegmental lymph node dissection and segment-specific dissection for very early-stage cancers. Long-term data maturation is still required when doing a head-to-head comparison between the two techniques, particularly in regard to long-term cost implications and survival implications, which have thus far not been demonstrated in minimally invasive surgery over open surgery (28, 29) and of course long-term morbidity and quality of life metrics.

## Immunobiological implications of lymph node resection

Murine tumor model data have shown that tumor-draining lymph nodes (TDLNs) are enriched for tumor-specific PD-1+ T cells, which are closely associated with PD-L1+ conventional dendritic cells (cDCs) (30). TDLN-targeted PD-L1 blockade induces enhanced antitumor T-cell immunity by seeding the tumor site with progenitor-exhausted T cells, resulting in improved tumor control. This data highlights the TDLN as a key site of immune regulation and control over the tumor microenvironment; thus, expanded dissection of the said nodes may lead to immune impairment due to less antigen exposure and immune priming. Further murine data were generated (31) from a metastatic lung cancer model where the primary subcutaneous tumors were resected with associated draining lymph nodes (dLN) remaining intact, completely resected, or partially resected. The median survival after surgery was significantly shorter with complete dLN resection at the time of surgery (49 days) compared to when lymph nodes remained intact (>88 days; *p* < 0.05). Survival was partially restored with incomplete lymph node resection and was CD8+ T-cell dependent. Similar observations were generated from Fransen's study (32) where surgical resection of TDLNs, but not contralateral lymph nodes, abolished therapy-induced tumor regressions and was associated with decreased immune infiltrate in the tumor microenvironment.

Application of these principles to the human setting was explored by Deng et al. (33) who retrospectively analyzed 144 patients with NSCLC who had recurred post-resection and stratified their outcomes based on TDLN count. Multivariate testing showed that a TDLN count of <16 (i.e., fewer nodes resected at the time of surgery) was associated with improved progression-free survival (PFS) in all cohorts [HR 0.26 (0.07–0.89), *p* = 0.03]. The prognostic benefit of a dLN count of ≤16 was more significant in immunotherapy alone, with no adjuvant treatment, pN1, female, and squamous carcinoma subgroups. A higher level of CD8+ central memory T cell (Tcm) within TDLNs was associated with improved PFS (HR: 0.235, 95% CI: 0.065–0.845, *p* = 0.027). Murine data from the syngeneic E0771 triple-negative breast cancer model showed that CD8+ T-cell priming occurs extratumorally in the dLN and the said antigenic priming is key to the survival of robust antitumor effector T-cell responses particularly in the context of checkpoint blockade (34). Progenitor-exhausted CD8+ T cells were abundant in uninvolved lymph nodes and mediate responses post-checkpoint blockade, but these responses were disrupted in metastatic lymph nodes (35).

Therefore, for patients planned for adjuvant immunotherapy, a precise rather than expanded lymphadenectomy strategy to preserve these key immunological sites upon which CD8+ priming is dependent may be important and worth consideration.

## Conclusions

There is no well-preserved standard on reproducible lymph node resection strategy to date. Systematic (MLND) vs. sampling was an early question that was only partially answered by ACOSOG Z0030. MLND was the preferred approach, but in early-stage (cT1) disease, there was a mixture of practice between sampling and MLND. Most studies do not include stage I disease only hence the controversy. Given the increased use of neoadjuvant immunotherapy, pathological interpretation is becoming more complex and necessary to determine factors such as MPR, pCR, and immunohistochemical features. Thus, it makes sense to perform MLND for robust pathological analysis and staging. The second question that remains largely unanswered is whether a selective strategy (lobe-specific) or a complete lymphadenectomy is performed. This is currently balanced on factors such as implications of TNM-9 staging and prognostication, lymph node immunobiology in the context of checkpoint blockade, and the incidence of postoperative complications. The implications of JCOG1413 are far-reaching and will hopefully serve to address these issues with impunity. In the era of minimally invasive surgery, whatever lymph node strategies are deemed most effective, the robotic approach will be able to effectively deliver with a low burden of morbidity and mortality.
